# Bis(hydrazin-1-ium) bis­(μ_2_-pyridazine-3,6-dicarboxyl­ato)bis­(aqua­lithiate) octa­aqua­bis­(μ_3_-pyridazine-3,6-dicarboxyl­ato)tetra­lithium

**DOI:** 10.1107/S1600536812007192

**Published:** 2012-02-24

**Authors:** Wojciech Starosta, Janusz Leciejewicz

**Affiliations:** a Institute of Nuclear Chemistry and Technology, ul. Dorodna 16, 03-195 Warszawa, Poland

## Abstract

The unit cell of the title compound, (N_2_H_5_)_2_[Li_2_(C_6_H_2_N_2_O_4_)_2_(H_2_O)_2_]·[Li_4_(C_6_H_2_N_2_O_4_)_2_(H_2_O)_8_], comprises two centrosymmetric complexes, one double negatively charged and one neutral, and two mono-protonated hydrazine cations. The anionic complex molecule is a dimer, built of a pair of symmetry-related pyridazine-3,6-dicarboxyl­ate ligands and a pair of Li^I^ ions, each coordinated by two *N*,*O*-chelating sites donated by a ligand mol­ecule and an aqua O atom at the apical position. The penta­coordination around the Li^I^ ions is partway between a trigonal–bipyramidal and a square-pyramidal arrangement. The two carb­oxy­lic acid groups of the ligand are deprotonated and one carboxyl­ate O atom of each group is not involved in the coordination, and this applies to both the anionic and the neutral complex. The neutral complex molecule is also composed of a pair of Li^I^ ions and a pair of ligand mol­ecules related by a centre of symmetry. They form a dimeric core in which the penta­coordination of the Li^I^ ions includes two *N*,*O*-bonding groups donated by two ligands and an aqua O atom. The penta­coordination is described as partway between a trigonal–bipyramidal and a square-pyramidal arrangement. The coordinated carboxyl­ate group is bidentate–bridging, forming with an Li(H_2_O)_3_ unit a neutral tetra­meric mol­ecule. The coordination of the tetra­coordinated Li^I^ ion shows a slightly distorted tetra­hedral geometry. An extended system of O—H⋯O and N—H⋯O hydrogen bonds contributes to the stability of the crystal structure.

## Related literature

For the crystal structures of Li^I^ complexes with pyridazine-3,6-dicarboxyl­ate and water ligands, see: Starosta & Leciejewicz (2010 ▶, 2011 ▶). The structure of a hydrazine adduct of pyridazine-3,6-dicarb­oxy­lic acid was reported by Starosta & Leciejewicz (2008 ▶).
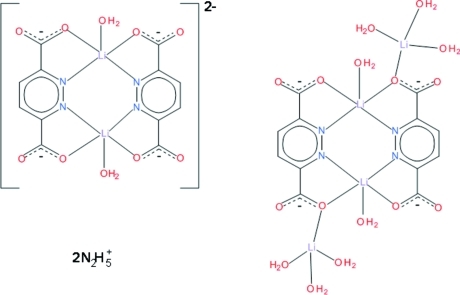


## Experimental

### 

#### Crystal data

(N_2_H_5_)_2_[Li_2_(C_6_H_2_N_2_O_4_)_2_(H_2_O)_2_]·[Li_4_(C_6_H_2_N_2_O_4_)_2_(H_2_O)_8_]
*M* *_r_* = 952.30
Triclinic, 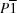

*a* = 7.0999 (14) Å
*b* = 7.2390 (14) Å
*c* = 22.608 (5) Å
α = 86.40 (3)°
β = 87.68 (3)°
γ = 61.49 (3)°
*V* = 1018.9 (3) Å^3^
*Z* = 1
Mo *K*α radiation
μ = 0.14 mm^−1^
*T* = 293 K
0.72 × 0.35 × 0.11 mm

#### Data collection

Kuma KM-4 four-circle diffractometer
Absorption correction: analytical (*CrysAlis RED*; Oxford Diffraction, 2008 ▶) *T*_min_ = 0.970, *T*_max_ = 0.985
5515 measured reflections
5058 independent reflections
3362 reflections with *I* > 2σ(*I*)
*R*_int_ = 0.090
3 standard reflections every 200 reflections intensity decay: 5.8%

#### Refinement

*R*[*F* ^2^ > 2σ(*F* ^2^)] = 0.054
*wR*(*F* ^2^) = 0.204
*S* = 1.05
5058 reflections
383 parameters
All H -atom parameters refined
Δρ_max_ = 0.63 e Å^−3^
Δρ_min_ = −0.41 e Å^−3^

### 

Data collection: *KM-4 Software* (Kuma, 1996 ▶); cell refinement: *KM-4 Software*; data reduction: *DATAPROC* (Kuma, 2001 ▶); program(s) used to solve structure: *SHELXS97* (Sheldrick, 2008 ▶); program(s) used to refine structure: *SHELXL97* (Sheldrick, 2008 ▶); molecular graphics: *SHELXTL* (Sheldrick, 2008 ▶); software used to prepare material for publication: *SHELXTL*.

## Supplementary Material

Crystal structure: contains datablock(s) I, global. DOI: 10.1107/S1600536812007192/kp2385sup1.cif

Structure factors: contains datablock(s) I. DOI: 10.1107/S1600536812007192/kp2385Isup2.hkl

Additional supplementary materials:  crystallographic information; 3D view; checkCIF report

## Figures and Tables

**Table 1:** Selected bond lengths (Å)

Li1—O11^i^	2.011 (4)
Li1—N11^i^	2.185 (4)
Li1—N12	2.209 (4)
Li1—O13	1.999 (4)
Li1—O15	1.961 (5)
Li2—O21	1.998 (4)
Li2—N22	2.175 (5)
Li2—O25	1.974 (4)
Li2—O24^ii^	2.023 (5)
Li2—N21^ii^	2.170 (4)
Li3—O21	1.951 (5)
Li3—O3	2.019 (5)
Li3—O2	1.926 (4)
Li3—O1	1.954 (4)

Symmetry codes: (i) 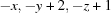; (ii) 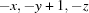.

**Table 2:** Hydrogen-bond geometry (Å, °)

*D*—H⋯*A*	*D*—H	H⋯*A*	*D*⋯*A*	*D*—H⋯*A*
O25—H252⋯O22^iii^	0.90 (4)	1.87 (4)	2.752 (3)	168 (3)
O1—H11⋯O23^iv^	0.74 (4)	2.10 (4)	2.832 (3)	173 (4)
N1—H1⋯O3	1.01 (4)	1.99 (4)	2.972 (3)	164 (3)
O2—H21⋯O23^v^	0.83 (3)	2.07 (3)	2.889 (3)	171 (3)
O2—H22⋯O11^vi^	0.81 (4)	1.88 (4)	2.691 (3)	172 (4)
N1—H2⋯O22^iii^	0.91 (3)	1.97 (4)	2.826 (3)	156 (3)
O3—H32⋯O24^ii^	0.94 (3)	1.71 (3)	2.635 (2)	165 (3)
N1—H3⋯O13	0.89 (4)	1.84 (4)	2.720 (3)	174 (3)
O15—H152⋯O12^vii^	0.86 (5)	1.92 (5)	2.755 (3)	162 (5)
O15—H151⋯O14^viii^	0.79 (4)	2.04 (4)	2.790 (3)	160 (4)
O3—H31⋯O14^ix^	0.71 (4)	2.14 (4)	2.833 (3)	165 (4)
O1—H12⋯O12^x^	0.83 (4)	2.06 (4)	2.839 (3)	155 (3)

Symmetry codes: (ii) 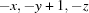; (iii) 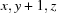; (iv) 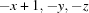; (v) 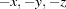; (vi) 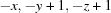; (vii) 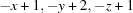; (viii) 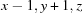; (ix) 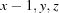; (x) 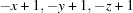.

**Fig. 1.**
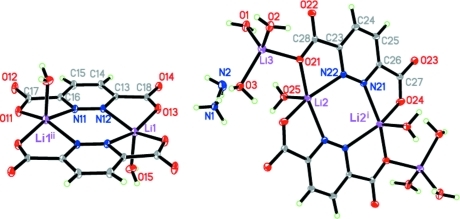
Structural units of the title compound with atom labelling scheme and 50% probability displacement ellipsoids. Symmetry code: i -x, -y + 1, -z; ii -x, -y + 2, -z + 1.

**Fig. 2.**
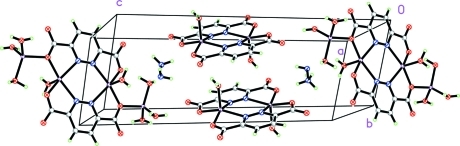
Crystal packing of the title compound.

## supplementary crystallographic information

### Comment

The structure of the original Li^I^ complex with pyridazine-3,6-dicarboxylate and water ligands was reported to consist of molecular ribbons in which Li^I^ ions are octahedrally coordinated by two fully deprotonated ligand molecules and two aqua O atoms are bridged by protons located in a centre of symmetry (Starosta & Leciejewicz, 2010). Removal of these protons by adding a few drops of hydrazine resulted in a two-dimensional catenated polymeric structure (Starosta & Leciejewicz, 2011). When few more drops of hydrazine were added to the aqueous solution of the original complex, crystals of a new compound with a triclinic centrosymmetric structure were identified. This structure is built of two mono protonated hydrazine cations, a centrosymmetric dimeric anion and a neutral centrosymmetric tetrameric molecule. The dimeric anion consists of pairs of symmetry related: Li^I^ ions, fully deprotonated ligand molecules and water O15 atom (Fig. 1). The Li1 ion, coordinated by two *N*,*O* bonding groups donated by two ligands and the aqua O15 atom shows transition from a distorted trigonal–bipyramidal geometry [with an equatorial plane composed of O13, N11^ii^ and O15 atoms with r.m.s. of 0.0059 (1) Å, the Li1 ion is 0.0119 (1) Å out of this plane, N12 and O11^ii^ atoms are at axial positions; symmetry code: ^i^ -*x*, -*y* + 1, -*z*; ^ii^ -*x*, -*y* + 2, -*z* + 1] to a square-pyramidal geometry [where the aqua O15 is at the apical position]. The pyridazine ring is planar (r.m.s. of 0.0017 (1) Å); carboxylate groups C17/O11/O12 and C18/O13/O14 make with it dihedral angles of 1.4 (1)° and 1.7 (1)°, respectively. An anionic dimer constitutes the core of the other complex molecule. The coordination of the Li2 ion can be described by transition from trigonal–bipyramidal arrrangement [N22, O24^i^ and O25 atoms form the equatorial plane, r.m.s. 0.0044 (1) Å, the Li2 ion is 0.0088 (1) Å out of the equatorial plane; O21 and N21^i^ are at the apices] to the square-pyramdidal one [with the water O25 at the apical position]. The pyridazine ring is planar [r.m.s. 0.0009 (1) Å]; the carboxylate C27/O21/O22 and C28/O23/O24 groups make with it dihedral angles of 5.9 (1)° and 0.7 (1)°, respectively. In contrast to the anion complex, the carboxylato O21 atom in the neutral complex molecule acts as bidentate bridging to a Li(H_2_O)_3_ group completing a tetranuclear molecule. The coordination environment of the Li3 ion formed by the O1, O2, O3 and O21 atoms is distorted tetrahedral. Pyridazine ring planes of the anion and the tetrameric molecule are inclined by an angle of 65.7 (1)° each to the other (Fig. 2). The observed Li—O and Li—N bond distances (Table 1) are close to those reported in two other Li^I^ complexes with the title ligand (Starosta & Leciejewicz, 2010, 2011). Bond distances in the protonated hydrazine cations are almost the same as those reported in the structure of an hydrazine adduct of the pyridazine-3,6-dicarboxylate acid (Starosta & Leciejewicz, 2008). An extended hydrogen bond system in which coordinated water molecules act as donors, carboxylate O atoms are as acceptors contributes to the stability of the structure (Table 2).

### Experimental

Single crystals of the compound obtained earlier (Starosta & Leciejewicz, 2010) were dissolved in warm water. Few drops of hydrazine were then added and the solution was stirred for 3 h without heating. Left to crystallize at room temperature, single-crystal blocks of the title compound were found after three days. They were washed with cold ethanol and dried in air.

### Refinement

Water and hydrazine H atoms were located in a difference map and refined isotropically, while H atoms attached to pyridazine-ring C atoms were located at calculated positions and treated as riding on the parent atoms with C—H = 0.93 Å and *U*_iso_(H) = 1.2*U*_eq_(C).

### Crystal data

**Table:**

(N_2_H_5_)_2_[Li_2_(C_6_H_2_N_2_O_4_)_2_(H_2_O)_2_]·[Li_4_(C_6_H_2_N_2_O_4_)_2_(H_2_O)_8_]	*Z* = 1
*M**_r_* = 952.30	*F*(000) = 492
Triclinic, *P*1	*D*_x_ = 1.552 Mg m^−^^3^
Hall symbol: -P 1	Mo *K*α radiation, λ = 0.71073 Å
*a* = 7.0999 (14) Å	Cell parameters from 25 reflections
*b* = 7.2390 (14) Å	θ = 6–15°
*c* = 22.608 (5) Å	µ = 0.14 mm^−^^1^
α = 86.40 (3)°	*T* = 293 K
β = 87.68 (3)°	Block, colourless
γ = 61.49 (3)°	0.72 × 0.35 × 0.11 mm
*V* = 1018.9 (3) Å^3^	

### Data collection

**Table:**

Kuma KM-4 four-circle diffractometer	3362 reflections with *I* > 2σ(*I*)
Radiation source: fine-focus sealed tube	*R*_int_ = 0.090
Graphite monochromator	θ_max_ = 30.1°, θ_min_ = 1.8°
profile data from ω/2θ scans	*h* = −10→9
Absorption correction: analytical (*CrysAlis RED*; Oxford Diffraction, 2008)	*k* = −9→10
*T*_min_ = 0.970, *T*_max_ = 0.985	*l* = −31→0
5515 measured reflections	3 standard reflections every 200 reflections
5058 independent reflections	intensity decay: 5.8%

### Refinement

**Table:**

Refinement on *F*^2^	Primary atom site location: structure-invariant direct methods
Least-squares matrix: full	Secondary atom site location: difference Fourier map
*R*[*F*^2^ > 2σ(*F*^2^)] = 0.054	Hydrogen site location: inferred from neighbouring sites
*wR*(*F*^2^) = 0.204	All H-atom parameters refined
*S* = 1.05	*w* = 1/[σ^2^(*F*_o_^2^) + (0.1491*P*)^2^ + 0.1309*P*] where *P* = (*F*_o_^2^ + 2*F*_c_^2^)/3
5058 reflections	(Δ/σ)_max_ < 0.001
383 parameters	Δρ_max_ = 0.63 e Å^−^^3^
0 restraints	Δρ_min_ = −0.41 e Å^−^^3^

### Special details

**Table:**

Geometry. All e.s.d.'s (except the e.s.d. in the dihedral angle between two l.s. planes) are estimated using the full covariance matrix. The cell e.s.d.'s are taken into account individually in the estimation of e.s.d.'s in distances, angles and torsion angles; correlations between e.s.d.'s in cell parameters are only used when they are defined by crystal symmetry. An approximate (isotropic) treatment of cell e.s.d.'s is used for estimating e.s.d.'s involving l.s. planes.
Refinement. Refinement of *F*^2^ against ALL reflections. The weighted *R*-factor *wR* and goodness of fit *S* are based on *F*^2^, conventional *R*-factors *R* are based on *F*, with *F* set to zero for negative *F*^2^. The threshold expression of *F*^2^ > σ(*F*^2^) is used only for calculating *R*-factors(gt) *etc*. and is not relevant to the choice of reflections for refinement. *R*-factors based on *F*^2^ are statistically about twice as large as those based on *F*, and *R*-factors based on ALL data will be even larger.

### Fractional atomic coordinates and isotropic or equivalent isotropic displacement parameters (Å^2^)

**Table:**

	*x*	*y*	*z*	*U*_iso_*/*U*_eq_	
O21	0.0159 (3)	0.2157 (2)	0.13963 (7)	0.0274 (4)	
N22	0.1040 (3)	0.2333 (3)	0.02543 (8)	0.0215 (4)	
O23	0.4250 (3)	−0.0353 (3)	−0.15562 (7)	0.0304 (4)	
O3	−0.1383 (3)	0.5109 (3)	0.25070 (8)	0.0318 (4)	
O25	0.2519 (3)	0.4707 (3)	0.11076 (8)	0.0336 (4)	
N21	0.1517 (3)	0.2519 (3)	−0.03187 (8)	0.0220 (4)	
C26	0.2698 (3)	0.0806 (3)	−0.06167 (9)	0.0203 (4)	
O22	0.1593 (3)	−0.1328 (2)	0.14161 (7)	0.0349 (4)	
O24	0.2395 (3)	0.3096 (3)	−0.14339 (7)	0.0375 (4)	
O2	−0.1801 (3)	0.0810 (3)	0.24722 (9)	0.0345 (4)	
O1	0.2959 (3)	0.0402 (3)	0.24937 (9)	0.0383 (4)	
C23	0.1751 (3)	0.0428 (3)	0.05183 (9)	0.0202 (4)	
C28	0.1110 (3)	0.0413 (3)	0.11678 (9)	0.0211 (4)	
C27	0.3165 (3)	0.1207 (3)	−0.12582 (10)	0.0233 (4)	
C25	0.3482 (4)	−0.1221 (3)	−0.03609 (10)	0.0274 (5)	
C24	0.2987 (4)	−0.1410 (3)	0.02252 (10)	0.0281 (5)	
Li2	−0.0167 (6)	0.4703 (6)	0.09159 (18)	0.0287 (8)	
Li3	−0.0032 (6)	0.2106 (6)	0.22601 (19)	0.0316 (8)	
H251	0.322 (5)	0.391 (5)	0.1328 (14)	0.037 (8)*	
H252	0.240 (6)	0.591 (6)	0.1232 (16)	0.062 (11)*	
O14	0.5966 (3)	0.5737 (3)	0.35267 (7)	0.0347 (4)	
O11	0.2464 (3)	0.9984 (3)	0.63816 (7)	0.0324 (4)	
N12	0.2675 (3)	0.8464 (3)	0.47199 (8)	0.0219 (4)	
O13	0.2417 (2)	0.7613 (3)	0.36274 (7)	0.0335 (4)	
O12	0.6015 (3)	0.8204 (3)	0.64806 (8)	0.0380 (5)	
N11	0.2686 (3)	0.8936 (3)	0.52789 (8)	0.0225 (4)	
C18	0.4299 (3)	0.6839 (3)	0.38075 (9)	0.0224 (4)	
C13	0.4527 (3)	0.7290 (3)	0.44400 (9)	0.0207 (4)	
O15	−0.0044 (3)	1.2454 (3)	0.38668 (10)	0.0399 (5)	
C16	0.4544 (3)	0.8219 (3)	0.55577 (9)	0.0215 (4)	
C17	0.4323 (3)	0.8865 (4)	0.61943 (9)	0.0243 (4)	
C14	0.6502 (3)	0.6519 (4)	0.47109 (11)	0.0291 (5)	
C15	0.6512 (3)	0.6996 (4)	0.52851 (11)	0.0303 (5)	
Li1	−0.0007 (6)	0.9775 (6)	0.40901 (18)	0.0282 (8)	
N1	0.1858 (3)	0.6592 (3)	0.25346 (9)	0.0293 (4)	
N2	0.3797 (4)	0.4809 (5)	0.23783 (15)	0.0537 (8)	
H11	0.361 (6)	0.048 (5)	0.2240 (16)	0.045 (10)*	
H4	0.451 (8)	0.543 (8)	0.225 (2)	0.088 (17)*	
H1	0.069 (7)	0.618 (6)	0.2603 (18)	0.071 (12)*	
H21	−0.243 (5)	0.073 (5)	0.2182 (15)	0.036 (8)*	
H22	−0.197 (6)	0.046 (6)	0.2811 (18)	0.055 (10)*	
H2	0.149 (6)	0.758 (5)	0.2232 (16)	0.051 (9)*	
H32	−0.197 (5)	0.591 (5)	0.2153 (15)	0.041 (8)*	
H3	0.211 (6)	0.694 (5)	0.2879 (16)	0.050 (9)*	
H152	0.108 (8)	1.237 (8)	0.369 (2)	0.099 (17)*	
H151	−0.100 (7)	1.343 (7)	0.3705 (18)	0.067 (12)*	
H31	−0.219 (7)	0.545 (6)	0.2730 (19)	0.061 (13)*	
H25	0.447 (5)	−0.246 (4)	−0.0595 (12)	0.029 (7)*	
H24	0.350 (4)	−0.279 (4)	0.0450 (12)	0.025 (6)*	
H15	0.774 (6)	0.660 (5)	0.5505 (17)	0.063 (11)*	
H14	0.775 (5)	0.573 (4)	0.4502 (13)	0.030 (7)*	
H12	0.348 (6)	0.042 (5)	0.2814 (17)	0.051 (10)*	
H5	0.447 (9)	0.442 (8)	0.270 (2)	0.103 (17)*	

### Atomic displacement parameters (Å^2^)

**Table:**

	*U*^11^	*U*^22^	*U*^33^	*U*^12^	*U*^13^	*U*^23^
O21	0.0347 (9)	0.0229 (7)	0.0225 (8)	−0.0123 (7)	0.0064 (6)	−0.0037 (6)
N22	0.0231 (8)	0.0228 (8)	0.0165 (8)	−0.0094 (7)	0.0007 (6)	−0.0012 (6)
O23	0.0292 (8)	0.0322 (8)	0.0228 (8)	−0.0088 (7)	0.0082 (6)	−0.0074 (6)
O3	0.0324 (9)	0.0361 (9)	0.0217 (9)	−0.0124 (7)	0.0042 (7)	−0.0010 (7)
O25	0.0336 (9)	0.0267 (8)	0.0353 (10)	−0.0098 (7)	−0.0041 (8)	−0.0022 (7)
N21	0.0215 (8)	0.0230 (8)	0.0177 (8)	−0.0078 (7)	0.0030 (6)	−0.0012 (6)
C26	0.0178 (9)	0.0235 (9)	0.0179 (9)	−0.0083 (7)	0.0010 (7)	−0.0026 (7)
O22	0.0538 (11)	0.0249 (8)	0.0243 (8)	−0.0179 (8)	0.0047 (8)	0.0002 (6)
O24	0.0456 (10)	0.0296 (8)	0.0219 (8)	−0.0065 (7)	0.0096 (7)	0.0020 (6)
O2	0.0310 (9)	0.0528 (11)	0.0251 (9)	−0.0248 (8)	0.0013 (7)	0.0000 (8)
O1	0.0292 (9)	0.0622 (12)	0.0263 (10)	−0.0241 (9)	0.0030 (8)	−0.0047 (9)
C23	0.0193 (9)	0.0228 (9)	0.0189 (9)	−0.0104 (8)	0.0011 (8)	−0.0011 (8)
C28	0.0232 (10)	0.0267 (10)	0.0153 (9)	−0.0134 (8)	0.0014 (7)	−0.0019 (8)
C27	0.0188 (9)	0.0268 (10)	0.0210 (10)	−0.0082 (8)	0.0027 (8)	−0.0032 (8)
C25	0.0287 (11)	0.0217 (10)	0.0246 (11)	−0.0061 (8)	0.0059 (9)	−0.0052 (8)
C24	0.0295 (11)	0.0228 (10)	0.0268 (11)	−0.0089 (8)	0.0038 (9)	0.0017 (8)
Li2	0.0308 (19)	0.0224 (17)	0.0280 (19)	−0.0087 (15)	0.0019 (15)	−0.0027 (15)
Li3	0.029 (2)	0.0324 (19)	0.033 (2)	−0.0146 (16)	0.0025 (16)	−0.0038 (16)
O14	0.0218 (8)	0.0426 (9)	0.0248 (9)	−0.0027 (7)	0.0027 (6)	−0.0083 (7)
O11	0.0196 (7)	0.0527 (10)	0.0222 (8)	−0.0143 (7)	0.0040 (6)	−0.0104 (7)
N12	0.0160 (8)	0.0299 (9)	0.0169 (8)	−0.0082 (7)	0.0005 (6)	−0.0048 (7)
O13	0.0183 (7)	0.0449 (10)	0.0254 (8)	−0.0040 (7)	−0.0036 (6)	−0.0120 (7)
O12	0.0205 (8)	0.0692 (13)	0.0245 (9)	−0.0206 (8)	−0.0019 (6)	−0.0093 (8)
N11	0.0157 (8)	0.0320 (9)	0.0186 (8)	−0.0103 (7)	0.0002 (6)	−0.0028 (7)
C18	0.0184 (9)	0.0268 (10)	0.0172 (9)	−0.0066 (8)	0.0004 (7)	−0.0039 (8)
C13	0.0163 (9)	0.0248 (9)	0.0196 (9)	−0.0085 (8)	0.0007 (7)	−0.0015 (7)
O15	0.0194 (8)	0.0408 (10)	0.0521 (12)	−0.0096 (7)	0.0010 (8)	0.0071 (9)
C16	0.0171 (9)	0.0310 (10)	0.0184 (9)	−0.0130 (8)	−0.0008 (7)	−0.0004 (8)
C17	0.0201 (10)	0.0371 (11)	0.0194 (10)	−0.0163 (9)	0.0010 (8)	−0.0035 (8)
C14	0.0144 (9)	0.0391 (12)	0.0269 (11)	−0.0062 (9)	0.0028 (8)	−0.0108 (9)
C15	0.0157 (10)	0.0441 (13)	0.0277 (11)	−0.0108 (9)	−0.0044 (8)	−0.0052 (10)
Li1	0.0137 (15)	0.0357 (19)	0.0287 (19)	−0.0063 (14)	0.0018 (14)	−0.0058 (15)
N1	0.0310 (10)	0.0318 (10)	0.0241 (10)	−0.0137 (8)	−0.0041 (8)	−0.0015 (8)
N2	0.0390 (14)	0.0484 (15)	0.0519 (17)	−0.0006 (12)	−0.0123 (12)	−0.0173 (12)

### Geometric parameters (Å, º)

**Table:**

Li1—O11^i^	2.011 (4)	O1—H11	0.74 (4)
Li1—N11^i^	2.185 (4)	O1—H12	0.83 (4)
Li1—N12	2.209 (4)	C23—C24	1.389 (3)
Li1—O13	1.999 (4)	C23—C28	1.521 (3)
Li1—O15	1.961 (5)	C25—C24	1.372 (3)
N11—Li1^i^	2.185 (4)	C25—H25	1.00 (3)
O21—C28	1.247 (3)	C24—H24	0.99 (3)
Li2—O21	1.998 (4)	Li3—H11	2.27 (4)
Li2—N22	2.175 (5)	O14—C18	1.242 (3)
Li2—O25	1.974 (4)	O11—C17	1.247 (3)
N21—Li2^ii^	2.170 (4)	O11—Li1^i^	2.011 (4)
O24—Li2^ii^	2.023 (5)	N12—N11	1.331 (3)
Li2—O24^ii^	2.023 (5)	N12—C13	1.336 (3)
Li2—N21^ii^	2.170 (4)	O13—C18	1.251 (2)
Li3—O21	1.951 (5)	O12—C17	1.252 (2)
Li3—O3	2.019 (5)	N11—C16	1.334 (2)
Li3—O2	1.926 (4)	C18—C13	1.518 (3)
Li3—O1	1.954 (4)	C13—C14	1.390 (3)
N22—C23	1.330 (2)	O15—H152	0.86 (5)
N22—N21	1.340 (2)	O15—H151	0.79 (4)
O23—C27	1.243 (3)	C16—C15	1.391 (3)
O3—H32	0.94 (3)	C16—C17	1.520 (3)
O3—H31	0.71 (4)	C14—C15	1.366 (3)
O25—H251	0.73 (3)	C14—H14	0.92 (3)
O25—H252	0.90 (4)	C15—H15	0.93 (4)
N21—C26	1.327 (3)	N1—N2	1.417 (3)
C26—C25	1.392 (3)	N1—H1	1.01 (4)
C26—C27	1.516 (3)	N1—H2	0.91 (3)
O22—C28	1.239 (2)	N1—H3	0.89 (4)
O24—C27	1.250 (3)	N2—H4	0.86 (5)
O2—H21	0.83 (3)	N2—H5	0.85 (5)
O2—H22	0.81 (4)		
			
C28—O21—Li3	116.39 (18)	O21—Li3—O3	107.8 (2)
C28—O21—Li2	118.44 (18)	O1—Li3—O3	113.9 (2)
Li3—O21—Li2	121.83 (18)	O2—Li3—H11	126.4 (9)
C23—N22—N21	119.55 (19)	O21—Li3—H11	86.0 (9)
C23—N22—Li2	109.87 (17)	O1—Li3—H11	18.2 (9)
N21—N22—Li2	129.03 (16)	O3—Li3—H11	113.5 (9)
Li3—O3—H32	103.6 (19)	C17—O11—Li1^i^	118.19 (19)
Li3—O3—H31	119 (3)	N11—N12—C13	119.78 (16)
H32—O3—H31	109 (4)	N11—N12—Li1	128.99 (17)
Li2—O25—H251	118 (2)	C13—N12—Li1	110.42 (16)
Li2—O25—H252	117 (2)	C18—O13—Li1	120.27 (18)
H251—O25—H252	103 (3)	N12—N11—C16	119.80 (19)
C26—N21—N22	119.84 (16)	N12—N11—Li1^i^	128.45 (16)
C26—N21—Li2^ii^	110.64 (17)	C16—N11—Li1^i^	110.42 (17)
N22—N21—Li2^ii^	128.61 (18)	O14—C18—O13	126.8 (2)
N21—C26—C25	122.8 (2)	O14—C18—C13	117.63 (18)
N21—C26—C27	115.22 (17)	O13—C18—C13	115.53 (19)
C25—C26—C27	122.0 (2)	N12—C13—C14	122.5 (2)
C27—O24—Li2^ii^	118.10 (18)	N12—C13—C18	114.71 (16)
Li3—O2—H21	112 (2)	C14—C13—C18	122.8 (2)
Li3—O2—H22	123 (3)	Li1—O15—H152	116 (3)
H21—O2—H22	124 (3)	Li1—O15—H151	125 (3)
Li3—O1—H11	106 (3)	H152—O15—H151	104 (4)
Li3—O1—H12	126 (2)	N11—C16—C15	122.6 (2)
H11—O1—H12	112 (4)	N11—C16—C17	114.32 (19)
N22—C23—C24	122.77 (19)	C15—C16—C17	123.10 (18)
N22—C23—C28	114.86 (19)	O11—C17—O12	126.4 (2)
C24—C23—C28	122.37 (17)	O11—C17—C16	116.52 (17)
O22—C28—O21	126.7 (2)	O12—C17—C16	117.0 (2)
O22—C28—C23	116.8 (2)	C15—C14—C13	117.7 (2)
O21—C28—C23	116.54 (17)	C15—C14—H14	122.2 (17)
O23—C27—O24	126.6 (2)	C13—C14—H14	120.0 (17)
O23—C27—C26	117.45 (18)	C14—C15—C16	117.62 (18)
O24—C27—C26	115.9 (2)	C14—C15—H15	125 (2)
C24—C25—C26	117.3 (2)	C16—C15—H15	117 (2)
C24—C25—H25	122.9 (15)	O15—Li1—O13	105.5 (2)
C26—C25—H25	119.6 (15)	O15—Li1—O11^i^	101.59 (18)
C25—C24—C23	117.70 (18)	O13—Li1—O11^i^	98.88 (19)
C25—C24—H24	123.4 (16)	O15—Li1—N11^i^	96.05 (18)
C23—C24—H24	118.9 (16)	O13—Li1—N11^i^	158.4 (2)
O25—Li2—O21	100.71 (18)	O11^i^—Li1—N11^i^	77.42 (14)
O25—Li2—O24^ii^	103.7 (2)	O15—Li1—N12	98.91 (18)
O21—Li2—O24^ii^	97.81 (19)	O13—Li1—N12	76.75 (14)
O25—Li2—N21^ii^	98.65 (18)	O11^i^—Li1—N12	159.5 (2)
O21—Li2—N21^ii^	160.6 (2)	N11^i^—Li1—N12	99.15 (17)
O24^ii^—Li2—N21^ii^	77.55 (14)	N2—N1—H1	110 (2)
O25—Li2—N22	99.76 (19)	N2—N1—H2	108 (2)
O21—Li2—N22	78.43 (14)	H1—N1—H2	110 (3)
O24^ii^—Li2—N22	156.5 (2)	N2—N1—H3	105 (2)
N21^ii^—Li2—N22	98.24 (17)	H1—N1—H3	107 (3)
O2—Li3—O21	105.5 (2)	H2—N1—H3	116 (3)
O2—Li3—O1	113.9 (2)	N1—N2—H4	99 (3)
O21—Li3—O1	102.8 (2)	N1—N2—H5	103 (4)
O2—Li3—O3	112.0 (2)	H4—N2—H5	93 (4)

Symmetry codes: (i) −*x*, −*y*+2, −*z*+1; (ii) −*x*, −*y*+1, −*z*.

### Hydrogen-bond geometry (Å, º)

**Table:**

*D*—H···*A*	*D*—H	H···*A*	*D*···*A*	*D*—H···*A*
O25—H252···O22^iii^	0.90 (4)	1.87 (4)	2.752 (3)	168 (3)
O1—H11···O23^iv^	0.74 (4)	2.10 (4)	2.832 (3)	173 (4)
N1—H1···O3	1.01 (4)	1.99 (4)	2.972 (3)	164 (3)
O2—H21···O23^v^	0.83 (3)	2.07 (3)	2.889 (3)	171 (3)
O2—H22···O11^vi^	0.81 (4)	1.88 (4)	2.691 (3)	172 (4)
N1—H2···O22^iii^	0.91 (3)	1.97 (4)	2.826 (3)	156 (3)
O3—H32···O24^ii^	0.94 (3)	1.71 (3)	2.635 (2)	165 (3)
N1—H3···O13	0.89 (4)	1.84 (4)	2.720 (3)	174 (3)
O15—H152···O12^vii^	0.86 (5)	1.92 (5)	2.755 (3)	162 (5)
O15—H151···O14^viii^	0.79 (4)	2.04 (4)	2.790 (3)	160 (4)
O3—H31···O14^ix^	0.71 (4)	2.14 (4)	2.833 (3)	165 (4)
O1—H12···O12^x^	0.83 (4)	2.06 (4)	2.839 (3)	155 (3)

Symmetry codes: (ii) −*x*, −*y*+1, −*z*; (iii) *x*, *y*+1, *z*; (iv) −*x*+1, −*y*, −*z*; (v) −*x*, −*y*, −*z*; (vi) −*x*, −*y*+1, −*z*+1; (vii) −*x*+1, −*y*+2, −*z*+1; (viii) *x*−1, *y*+1, *z*; (ix) *x*−1, *y*, *z*; (x) −*x*+1, −*y*+1, −*z*+1.
